# Genetic Characteristic and Global Transmission of Influenza A H9N2 Virus

**DOI:** 10.3389/fmicb.2017.02611

**Published:** 2017-12-22

**Authors:** Mingda Hu, Yuan Jin, Jing Zhou, Zhisong Huang, Beiping Li, Wei Zhou, Hongguang Ren, Junjie Yue, Long Liang

**Affiliations:** Laboratory of Genetic Engineering, Beijing Institute of Biotechnology, Beijing, China

**Keywords:** Influenza A H9N2 virus, transmission, phylogeography, global, lineage

## Abstract

The H9N2 virus has been demonstrated to donate its genes to other subtypes of influenza A virus, forming new reassortant virus which may infect human beings. Understanding the genetic characteristic and the global transmission patterns of the virus would guide the prevention and control of potentially emerging avian influenza A virus. In this paper, we hierarchically classified the evolution of the H9N2 virus into three main lineages based on the phylogenetic characteristics of the virus. Due to the distribution of sampling locations, we named the three lineages as Worldwide lineage, Asia-Africa lineage, and China lineage. Codon usage analysis and selective positive site analysis of the lineages further showed the lineage-specific evolution of the virus. We reconstructed the transmission routes of the virus in the three lineages through phylogeography analysis, by which several epicenters for migration of the virus were identified. The hierarchical classification of the lineages implied a possible original seeding process of the virus, starting from the Worldwide lineages to the Asian-Africa lineages and to the China lineages. In the process of H9N2 virus global transmission, the United States was the origin of the virus. China Mainland, Hong Kong SAR, Japan, and Korea were important transfer centers. Based on both the transmission route and the distribution of the hosts in each lineage, we concluded that the wild birds' migration has contributed much to the long-distance global spread of the virus, while poultry trade and people's lifestyle may have contributed to the relatively short-distance transmission in some areas of the Asia and Africa.

## Introduction

The H9N2 influenza virus was isolated for the first time from turkeys in Wisconsin in 1966 (Homme and Easterday, 1970). Since then, H9N2 avian influenza viruses have been detected in domestic poultry and wild birds in North America, then detected from multiple avian species of Europe, Africa, Asia, and the Middle East. Now, the H9N2 avian influenza virus is widely distributed in different regions of the world and has become one of the dominant subtypes of influenza virus circulating in poultry and wild birds (Song et al., 2011).

H9N2 virus occasionally expands its host range to mammalian species. H9N2 virus infection in pig farms has been confirmed in Hong Kong and the mainland of China (Peiris et al., 2001; Xu et al., 2004). More importantly, several infectious cases in humans exhibiting mild respiratory disease have been reported since 1997 from Hong Kong and other provinces of China (Peiris et al., 1999; Butt et al., 2005).

In addition, prior phylogenetic analysis showed that the influenza A H9N2 viruses have contributed to some zoonotic spillover events by providing some internal gene segments to the reassortment of the cross-species virus (Guan et al., 2000; Dalby and Iqbal, 2014; Jin et al., 2017; Wu et al., 2017). In the global H5N1 outbreak started in Hong Kong 1997, H9N2 virus was demonstrated to have donated its six internal genes to highly pathogenic avian influenza (HPIV) H5N1 viruses (Subbarao and Cox, 1998; Guan et al., 2000). The novel avian influenza A H7N9 virus which caused the 2013 H7N9 outbreak was also found to be a reassortant virus with all the six internal genes from avian influenza A (H9N2) viruses (Gao et al., 2013). Except for H7N9, the internal genes of H10N8 also originated from H9N2 (Liu et al., 2013, 2014; Qi et al., 2014). The co-circulation of H9N2 viruses with other subtypes of influenza A virus may increase the risk of forming new reassortant viruses that could overcome the host barriers and infect human.

In our previous work (Yuan et al., 2014), the migration patterns of H9N2 virus circulated in China have been studied using a Bayesian phylogeography approach, yet the genetic diversity and global transmission of the H9N2 virus remains poorly understood (Butler, 2012).

The formation of genetic diversity and worldwide distribution of virus was a complex dynamic process driven by both internal and environmental forces (Ren et al., 2016), and the reconstruction of the evolution history could be both rational and computational challenging. In this paper, we performed a comprehensive genetic analysis of all the H9N2 virus with available genome sequences, aiming at providing an overall view of the global ecological dynamics of the H9N2 virus. Interestingly, the H9N2 virus sampled around the world exhibited clearly lineage-specific evolutionary characteristics. The transmission of the virus was shown to be consisting of hierarchical seeding and local persisting, driven by several transmission centers at each level.

## Materials and methods

### Sequence data preparation and alignment

All hemagglutinin (HA) gene and neuraminidase (NA) sequences of H9N2 viruses in this research were downloaded from NCBI Influenza Virus Resource (Bao et al., 2008). To reduce the number of sequences, sequences <95% full length were removed, and the resulting sequences were clustered using CD-HIT v4.6 (Li et al., 2001) with a threshold level of 0.95. Our sequences dataset included 2127 HA sequences and 1591 NA sequences. The coding region of the sequences were aligned using MAFFT v7.058 (Katoh et al., 2002) and then were inspected manually according to the amino acid sequences using Mega v5.05 (Tamura et al., 2011).

In order to reduce the computational complexity in the phylogeography analysis, we re-sampled some sequences from each cluster, and took one or two strains per year, per location, and per host, resulting one resampling dataset (Supplementary Table 1).

### Phylogeny and phylogeography reconstruction

Bayesian analysis was conducted with MrBayes v3.2 (Huelsenbeck and Ronquist, 2001), and by using 10 million generations and sample frequencies to obtain standard deviation of split frequencies below 0.01. Bayesian posterior probabilities were calculated from the consensus of 18,000 trees after excluding the first 2,000 trees as burn-in.

We also used a root-to-tip regression of genetic distances against sampling time in the program TempEst v1.5 (Rambaut et al., 2016) to find the best-fitting root of MrBayes trees.

We inferred time-scaled phylogenies by Bayesian Markov Chain Monte Carlo (MCMC) sampling using BEAST v1.8.0 (Drummond and Rambaut, 2007). The SRD06 codon position model and the uncorrelated log-normal relaxed clock model under a Constant Size coalescent tree prior in the MCMC simulations (Drummond et al., 2006; Shapiro et al., 2006; Rambaut et al., 2014) was used to elucidate the population dynamics of H9N2 viruses.

To infer ancestor location and migration events, we first used MrBayes v3.2.3 (Huelsenbeck and Ronquist, 2001) to build the whole evolutionary tree of H9N2. Then we divided the sequences into several lineages according to the tree, and we found each lineage had a relatively independent migration pathway. So that, we grouped sequences in each lineage into several localities.

In order to avoid the error caused by data bias, we re-sampled the sequence data in accordance with the following rules, collecting a sequence per year per location. Then the spatial location reconstruction and viral migration were estimated using the discrete Bayesian stochastic search variable selection (BSSVS) model (Lemey et al., 2009).

For each lineage, we performed 40 replicates, and in each replicate, we performed three to seven independent runs for 100 million generations with sampling every 10,000 steps to get a stable result. Convergence and effective sampling size (ESS) of estimates were assessed by visual inspection using Tracer v1.6 (Rambaut et al., 2014). Multiple chains were then combined after a 10% burn-in using LogCombiner v1.8.0 include in the BEAST package. The maximum clade credibility (MCC) trees with temporal and spatial annotation were summarized with a 10% burn-in removed using TreeAnnotator v1.8.0 in the BEAST package and presentation figures were generated with FigTree v1.4.2 (Rambaut, 2014).

### The COA analysis of codon usage bias

Codon usage of the influenza viruses was examined in this study using relative synonymous codon usage (RSCU) values (Sharp et al., 1986) and Correspondence Analysis (COA) (Greenacre, 1984). COA is a type of multivariate analysis that allows a geometrical representation of the sets of rows and columns in a dataset (Wong et al., 2010).

COA based on RSCU values appears to be an effective tool to reveal evolutionary trends and to classify influenza sequences by host and subtype. RSCU values from a novel sequence can be mapped to the existing axes of a COA to reveal the relationship of that sequence to existing groups as shown by the validation tests performed in this work. This method also allows prompt visual identification of viral reassortants or zoonotic transfer in influenza genes without the need to perform extensive computations. We used the codonW 1.4.2^1^ software to do COA analysis (Wong et al., 2010).

### Selection pressure analysis

Selection analyses of H9N2 strains from each lineage were performed using the Datamonkey web-server (www.datamonkey.org; Pond and Frost, 2005). In addition to the Single-likelihood ancestor counting (SLAC), Fixed effects likelihood (FEL), Mixed Effects Model of Evolution (MEME), and Fast Unconstrained Bayesian Approximation for inferring selection (FUBAR) methods were also proposed to estimate the selection pressure (Supplementary Tables 9, 10).

### Availability of supporting data

All gene sequences of H9N2 viruses used in this study were downloaded from NCBI Influenza Virus Resource. Accession numbers of hemagglutinin gene sequences can be found in additional files.

## Results

### Phylogenetic characteristics of H9N2 virus

**Figure 1:**
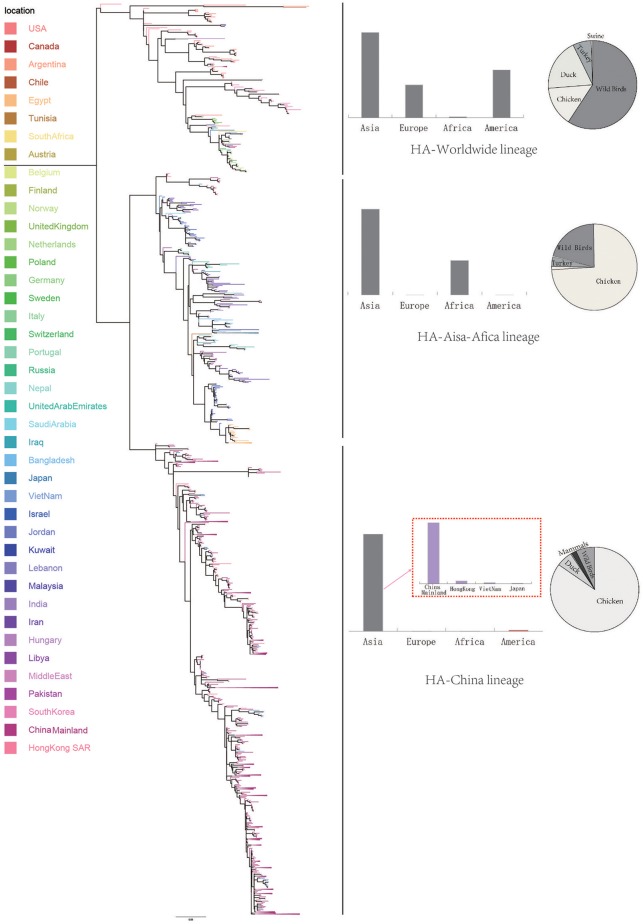
Bayesian phylogenetic tree of the hemagglutinin (HA) gene of avian influenza A H9N2. The branches are colored according to the location of their nodes. Histograms on the right side of the phylogenetic tree show the geographical distribution of each lineage. The sequences in HA lineage I were from Asia, Europe, Africa, and America, so we named HA lineage I as HA-World lineage. The sequences in HA lineage II were from Asia and Africa, so we named HA lineage II as HA-Asia-Africa lineage. The sequences in HA lineage III were only from Asia, and if we were more precise, we would find most sequences in lineage III were from Hong Kong and other provinces in China. So, we named HA lineage III as HA-China lineage.

**Figure 2:**
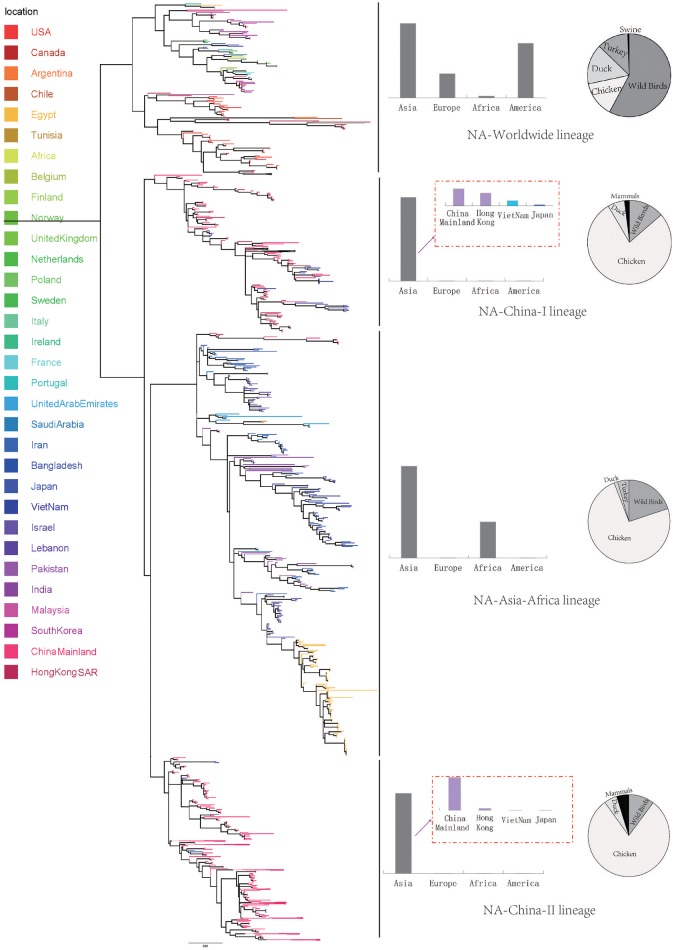
Bayesian phylogenetic tree of the neuraminidase (NA) gene of avian influenza A H9N2. The branches are colored according to the location of their nodes. Histograms on the right side of the phylogenetic tree show the geographical distribution of each lineage. The sequences in NA lineage I were from Asia, Europe, Africa and America, so we named NA lineage I as NA-World lineage. The sequences in NA lineage II were only from Asia, and if we were more precise, we would find most sequences in lineage II were from Hong Kong and other provinces in China. So, we named NA lineage II as NA-China-I lineage. The sequences in NA lineage III were from Asia and Africa, so we named NA lineage III as NA-Asia-Africa lineage. The sequences in HA lineage IV were only from Asia, and if we were more precise, we would find most sequences in lineage IV were from Hong Kong and other provinces in China. So, we named NA lineage IV as NA-China-II lineage.

We retrieve all available HA sequences and NA sequences of H9N2 viruses from GenBank (Bao et al., 2008), resulting a genome set consisting of 2127 HA sequences and 1591 NA sequences. Based on this genome set, we constructed two genealogical trees of H9N2 virus for the HA and NA gene segments. The HA and NA phylogenetic trees could be clearly classified into three and four genetic lineages respectively (Figures 1, 2).

Due to the distribution of sampling locations of the strains in each lineage, we named the three lineages in the HA tree as HA-Worldwide lineage, HA-Asia-Africa lineage and HA-China lineage respectively. Accordingly, there are NA-Worldwide lineage, NA-Asia-Africa lineage in the NA tree. The corresponding China-lineage in the NA tree was divided into two separate lineages namely NA-China-I lineage and NA-China-II lineage.

In the HA-Worldwide lineage, the virus was widely distributed on several continents in the world. The hosts of the virus in this lineage mainly consisted of wild birds, with a minority of chicken, ducks and swine. The virus in the HA-Asia-Africa lineage were mainly sampled from East Asia, Middle East Asia, Southeast Asia, and Africa. Some strains sampled from Hong Kong SAR was predicted to be rooting the HA-Asia-Africa lineage, which indicated that the Hong Kong SAR may have been the origin of this lineage. Poultry hosts acted as major hosts of the HA-Asia-Africa lineage, while the wild birds took a minority of the host distribution, which showed a reverse host distribution pattern compared with the HA-Worldwide lineage. Although the HA-China lineage took the smallest geographical distribution range in all the three lineages of the HA tree, its host distribution showed the most diversity. Poultry were major hosts in the HA-China lineage and the rest consisted of wild birds, swine, canine, and equine etc. It should be noted that not all the viruses in the HA-China lineage were sampled from China, there were a small portion of the viruses were from Japan and Viet Nam.

Interestingly, if we treated the NA-China-I lineage and the NA-China-II lineage as a whole, the location and host distributions of all the lineages in the NA tree exhibited similar patterns as in the HA tree respectively. This implied that the H9N2 virus might have transmitted globally with the two surface proteins “bounded.”

### Codon usage patterns and selection pressure analysis of H9N2 virus

To determine the trend in codon usage variation among the coding sequences of different H9N2 lineages, we performed a Correspondence Analysis (COA) (Wong et al., 2010) on both HA and NA proteins. The analysis was used to identify the systematic relationship between variables. Additionally, it simplifies complex data to deliver different strains or genes in multidimensional space (Butt, 2014). The COA was performed on the relative synonymous codon usage (RSCU) values for each strain and determined allocation in the first three principal axes of the plan.

**Figure 3:**
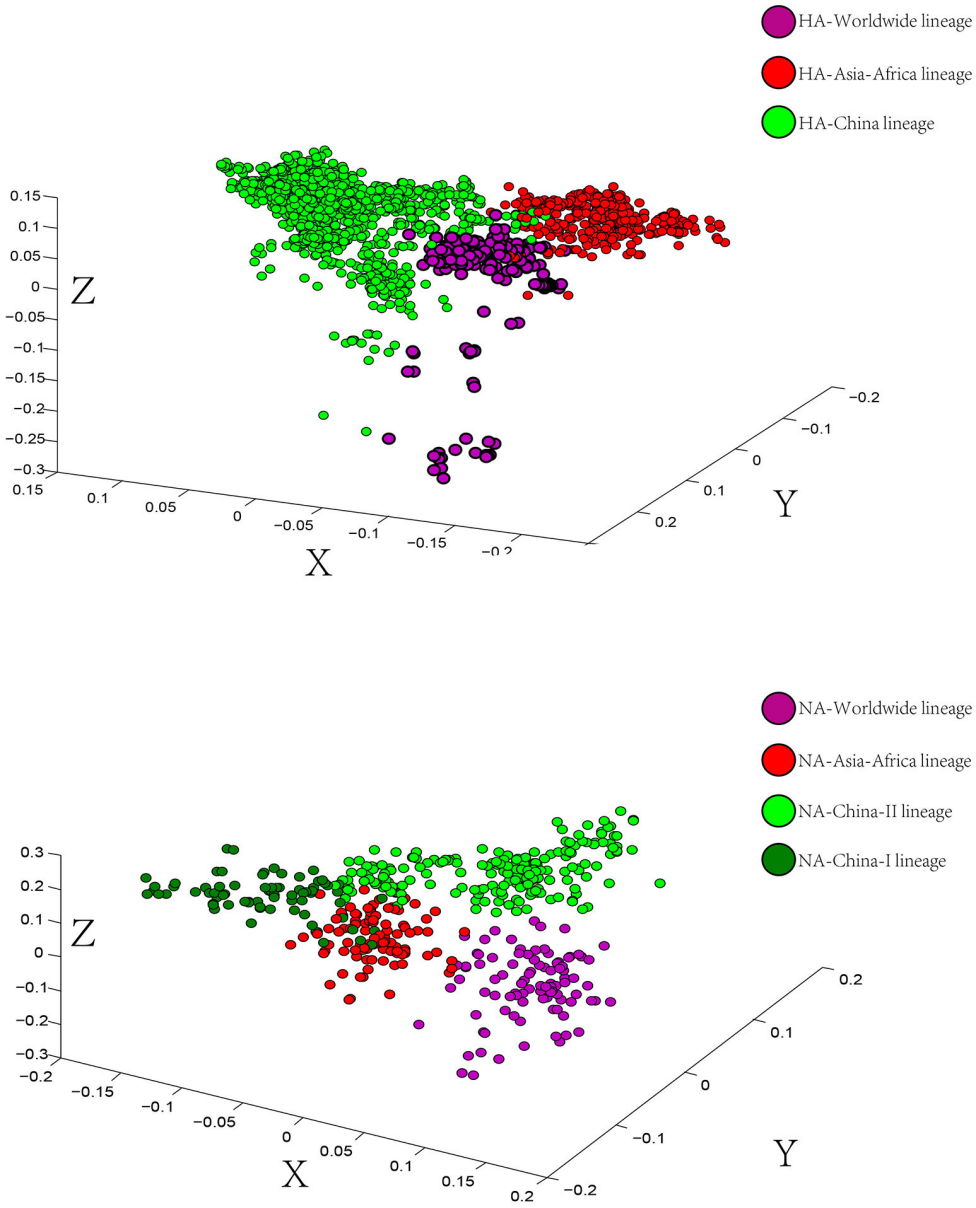
Correspondence Analysis for the hemagglutinin (HA) and neuraminidase (NA) genes of avian influenza A H9N2. Each viral gene is displayed in a 3-dimensional representation. The axes are in arbitrary scales generated by the COA and the weight of each codon in these axes varies in different segments. The circles were colored according to the lineage of their sequences. Circles of the same color have a tendency to be distributed together. The X axis is the first principal axes, Y axis is the second one and Z axis is the third principal axes.

The scattered data in principal axis represents different strains of viruses in different lineages and their relationship with each other (Figure 3). According to the COA results, we can see that viruses belonging to the same lineage can be clustered into the same group with obscure boundaries in the three-dimensional space for both the HA and the NA gene segments. The one-to-one mapping of the lineages in the phylogenetic trees and the codon usage patterns implied a lineage-specific codon usage bias in the H9N2 virus.

Using different codon-based and branch-site approaches, we detected a number of codons subjected to positive pressure in each lineage of H9N2 virus (Tables 1, 2). The analysis was done using the DataMonkey web-server (www.datamonkey.org; Takakuwa et al., 2013). We found that different number of codons in different lineages of both HA and NA trees were detected as positively selected by at least two methods (Tables 1, 2). There were 6 positive selected codons in the HA-Worldwide lineage, 8 in the HA-Asia-Africa lineage and 12 in the HA-China lineage. As for NA lineages, the NA-Worldwide lineage had 2 positive selected codons. The NA-Asia-Africa lineage had 8 positive selected codons and the NA-China lineage had 8 positive selected codons. There existed differences of the number of positive selected codons in different lineages. Furthermore, the positions of these codons were also mostly different among lineages. It seemed that different lineages in the same gene segment might have been facing different levels of selection pressures.

**Table 1:** Positively selected sites (at least two methods) of HA protein.

**HA lineages**	**Number of sites under differential selection at least two methods**	**Positively selected codon sites**
Worldwide lineage	6	3,13, 38, 40, 287, 337
Asia-Africa lineage	8	42, 168, 198, 201, 204, 234, 282, 537
China lineage	12	3, 4, 15, 149, 168, 182, 198, 234, 353, 381, 556, 557

**Table 2:** Positively selected sites (at least two methods) of NA protein.

**NA lineages**	**Number of sites under differential selection at least two methods**	**Positively selected codon sites**
Worldwide lineage	2	358, 414
Asia-Africa lineage	8	19, 42, 77, 356, 403, 416, 432, 468
China-I lineage	5	73, 81, 149, 296, 384
China-II lineage	3	9, 249, 468

### Phylogeography reconstruction and global transmission of H9N2 virus

To explore the transmission patterns of H9N2 virus, we performed phylogenetic analysis of each lineage of both HA and NA trees. Through a Bayesian phylogeography framework, we reconstructed phylogeographic MCC (maximum clade credibility) trees with time-scale and inferred ancestral locations of each branch using sequences' sampling collection dates and locations (Yuan et al., 2014). Furthermore, in order to gain insight into the spatial temporal dynamics of the geographic diffusion process of the H9N2 virus, we transformed the spatial estimates annotated in the MCC trees into the spreading network on the actual map.

**Figure 4:**
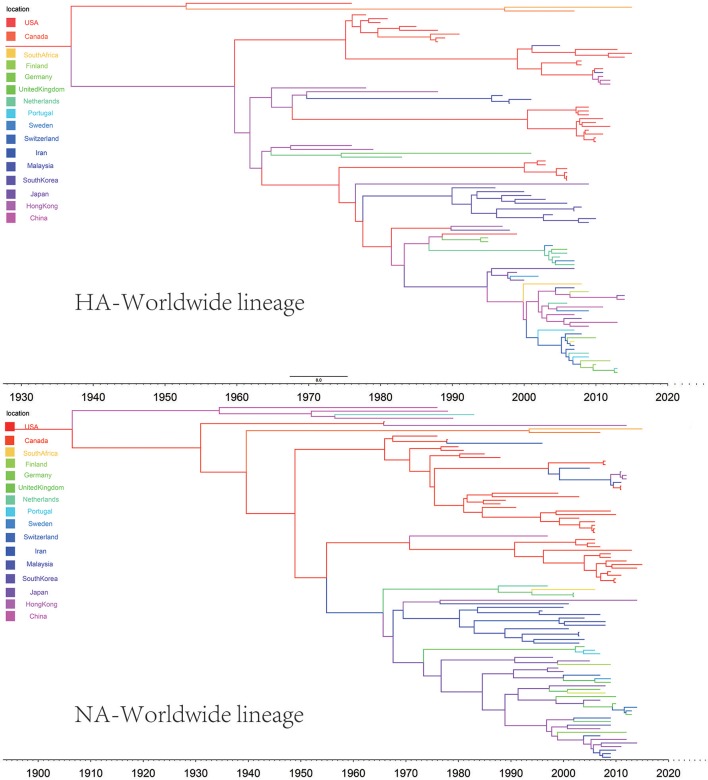
Maximum clade credibility (MCC) phylogenies for the hemagglutinin (HA) and neuraminidase (NA) genes of avian influenza A H9N2 in the Worldwide lineage. The branches are colored according to the most probable ancestor location of their descendent nodes. The scale bar at the bottom indicates the years before the most recent sampling time.

In the genealogical trees of HA-Worldwide lineage and NA-Worldwide lineage of H9N2 virus (Figure 4), we found that after the originating in the United States, the virus spread to Hong Kong SAR where it deployed active evolution and started to spread further (Supplementary Tables 2, 3). In the corresponding spreading networks of the Worldwide lineages of HA and NA (**Figure 7**), large geographical transmission distances, like from USA to Japan, Japan to European countries, were observed. The higher transmission connections of China (including Hong Kong SAR) and Japan suggested that these areas may have acted as transfer centers for the global migration of the H9N2 virus. The spreading routes largely coincided with migratory flyways of birds, which indicated an important role of the migratory birds in carrying and seeding the virus globally.

**Figure 5:**
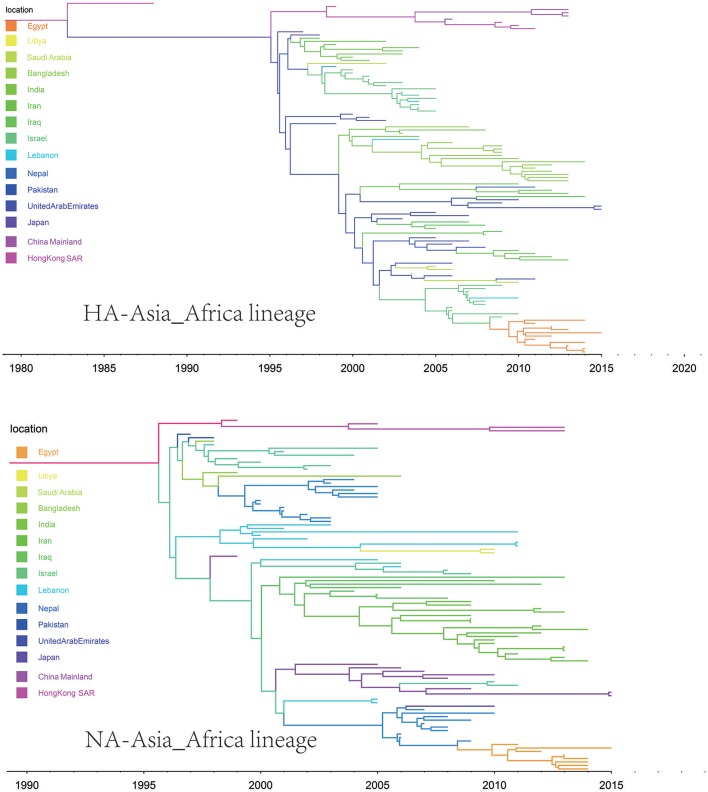
Maximum clade credibility (MCC) phylogenies for the hemagglutinin (HA) and neuraminidase (NA) genes of avian influenza A H9N2 in the Asia-Africa lineage. The branches are colored according to the most probable ancestor location of their descendent nodes. The scale bar at the bottom indicates the years before the most recent sampling time.

The origins of both HA-Asia-Africa lineage and NA-Asia-Africa lineage were allocated in Hong Kong SAR around the 1990s in the MCC trees (Figure 5). The virus then spread from Asia to the Middle East, through which the virus reached the North Africa (Figure 8; Supplementary Tables 4, 5). Surprisingly, the virus in the Asia-Africa lineages seemed to prefer to circulate in a horizontal manner in a relatively narrow latitude range, which may be related to the ecological tropism of the virus. Unlike the long-distance migration in the Worldwide lineages, the virus in the Asia-Africa lineages tended to be transmitted between neighboring countries. This was partially because the hosts of the Asia-Africa lineages were mainly poultry with some falcons and parakeets, which do not usually travel long distances like the migratory birds.

**Figure 6:**
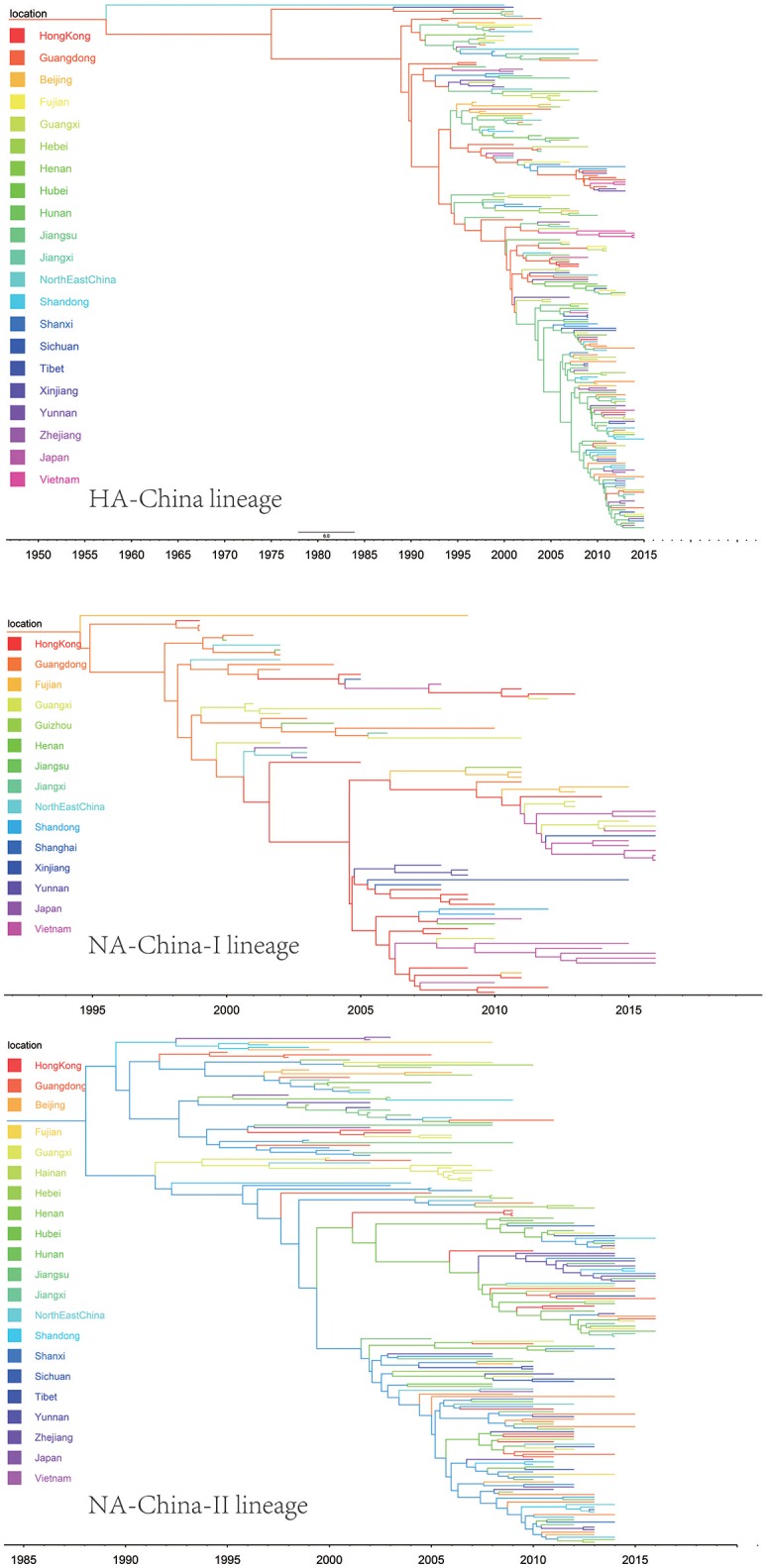
Maximum clade credibility (MCC) phylogenies for the hemagglutinin (HA) and neuraminidase (NA) genes of avian influenza A H9N2 in the China lineage. The branches are colored according to the most probable ancestor location of their descendent nodes. The scale bar at the bottom indicates the years before the most recent sampling time.

The H9N2 virus in China formed a relatively independent and complex evolutionary ecology. In our previous work (Yuan et al., 2014), we have thoroughly analyzed the origin, migration patterns and demography history of H9N2 virus circulating in China. To keep the integrity of this work, we reconstructed the phylogeographic trees and spreading networks of the virus in our China lineages for both HA and NA gene segments (Figures 6, **9**; Supplementary Tables 6–8). Although a different dataset was used in the analysis, almost consistent conclusions were obtained. For the NA gene segments, the China lineage was separated into two sub-lineages (NA-China-lineage-I and NA-China-lineage-II) by the NA-Asia-Africa lineage. We analyzed the two sub-lineages separately. The result shows that Guangdong and Shanghai were the epicenters for each of the sub-lineage. This was slightly different from our previous work in which all the H9N2 virus in China was treated as a whole. Furthermore, the China lineage in this paper included some strains sampled from Japan and Viet Nam. Since most of the H9N2 virus in China were hosted in poultry, live poultry trade in China may have promoted the migration of the virus.

## Discussion

The hierarchical division of the H9N2 virus into three main lineages in this paper offers a new view of the transmission patterns of the virus. We systematically analyzed the relatively independent genetic evolution and interrelated transmission of the virus in each lineage. It seems that the geographical isolation and the host migration of the H9N2 virus have jointly led to the global ecology of the virus.

Through the codon usage analysis and positive selection site analysis, we noticed codon usage patterns implied a lineage-specific codon usage bias in the H9N2 virus and different lineages have been facing different levels of selection pressures, which further revealed the unique genetic characteristic of each lineage, which may be related to the selective pressures given by the hosts and the evolutionary background of the virus in each lineage. Interestingly, Worldwide lineage had the largest geographic area, but the number of positive selected codons in Worldwide lineage was the smallest. Oppositely, China lineage had the smallest geographic area and largest number of positive selected codons. The differences between Worldwide lineage and China lineage suggested that large geographic areas of transmission could provide a more diverse environment for survival, and selective pressures for the virus might be at a relatively low level.

The hierarchical classification of the lineages in both the HA and NA implied a possible original seeding process of the virus, starting from the Worldwide lineage to the Asian-Africa lineage and to the China lineage. While the transmission of the virus from each lineage showed its specific pattern, there were overlaps in the transmission routes, where the interchange of genetic variation may have happened (Greenacre, 1984; Wong et al., 2010).

**Figure 7:**
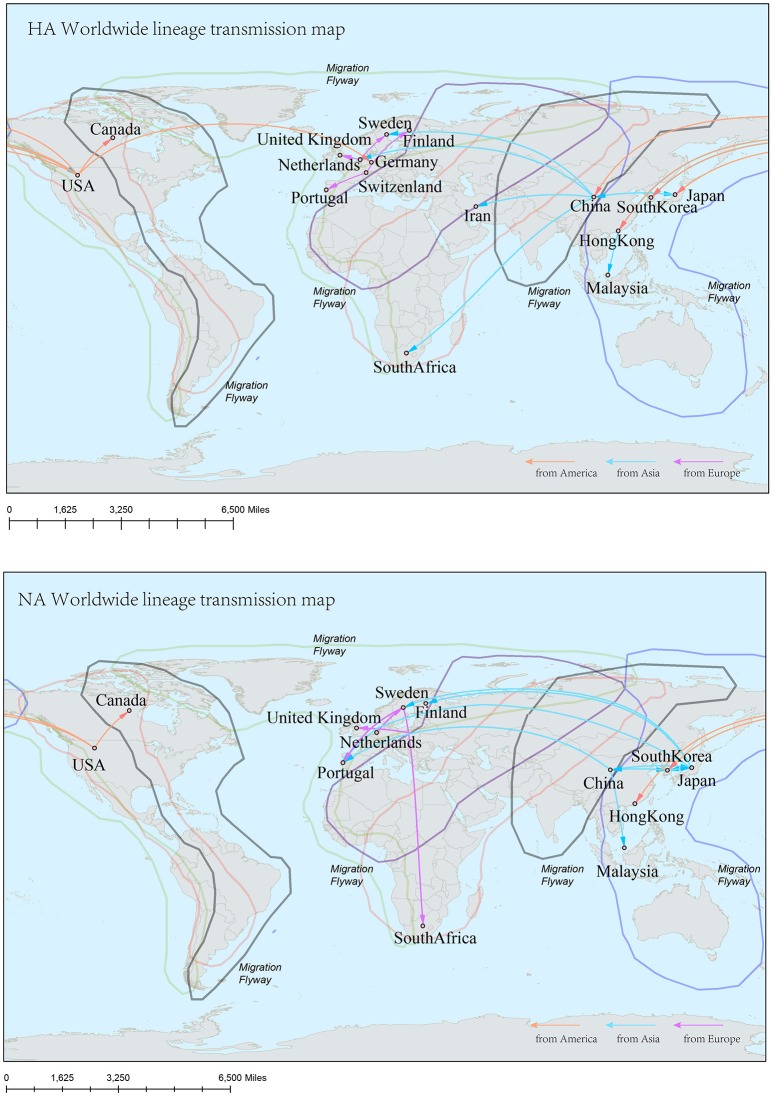
Spatial migration network of H9N2 virus geographic dispersal in the Worldwide lineage. Arrows represent direction of movement, and the arrow color is proportional to starting area. The migration event was starting from America to Asia, and then from Asia to Europe and Africa. The general direction of propagation was from east to west.

**Figure 8:**
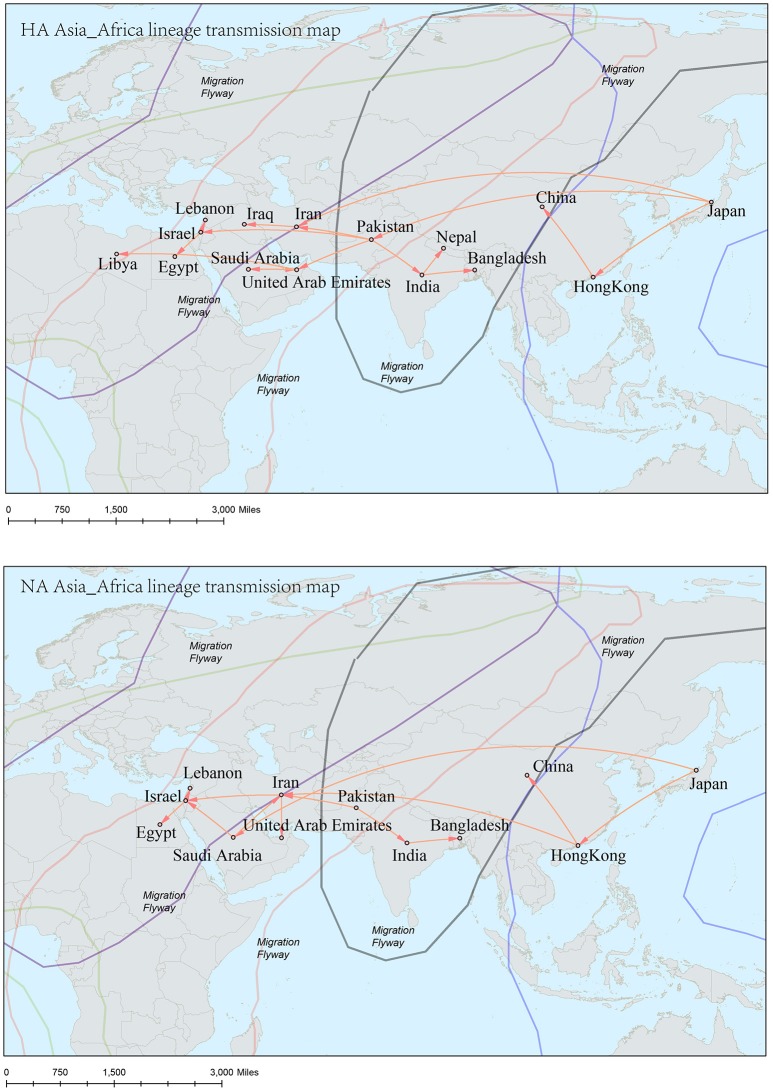
Spatial migration network of H9N2 virus geographic dispersal in the Asia-Africa lineage. Arrows represent direction of movement. The migration event was starting from Hong Kong and Japan, and spreading to East Asia, the Middle East, West Asia and Africa. The general direction of propagation was from east to west.

**Figure 9:**
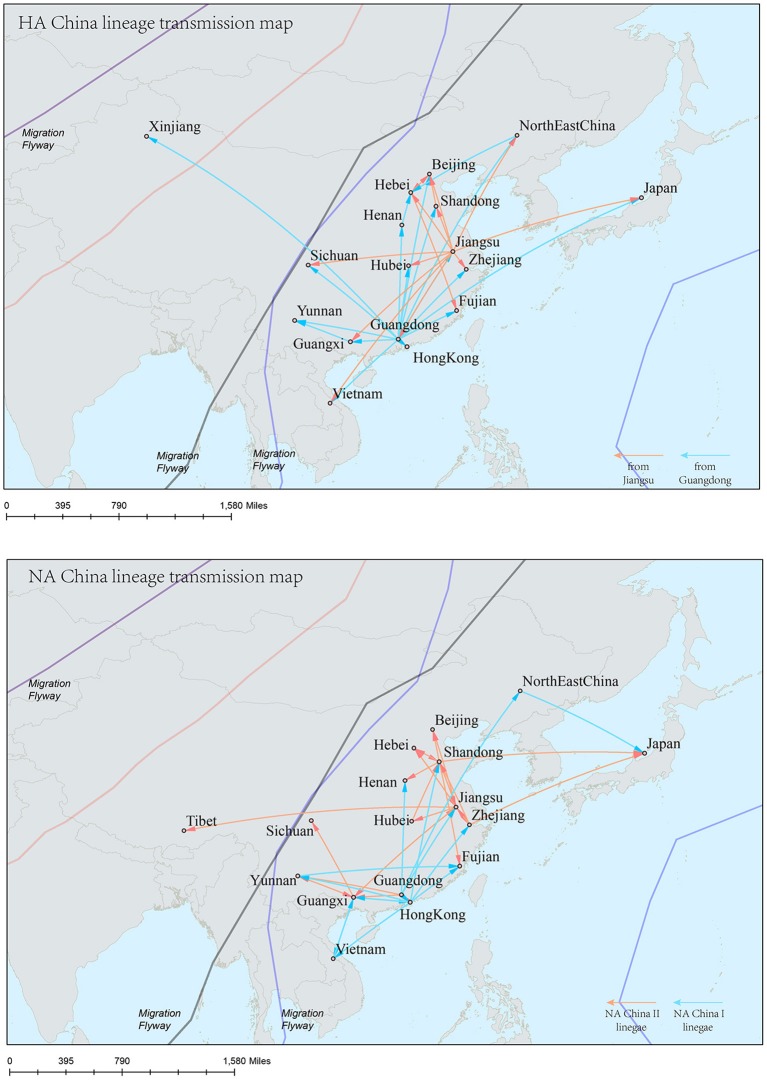
Spatial migration network of H9N2 virus geographic dispersal in the China lineage. Arrows represent direction of movement, and the arrow color in HA-China lineage migration network is proportional to starting area. As for NA-China lineage the orange color represents NA-China-II lineage, and the blue color represents NA-China-I lineage. The migration event was starting from Guangdong and Jiangsu Province, and spreading to the other areas in China, Viet Nam, and Japan.

Reconstruction of the transmission routes of the H9N2 virus based on the Bayesian phylogeography approach offered a panorama for the movement of the virus^1^. Higher connection degrees of the locations in the transmission network means that these places may have been the epicenters for the spreading of the virus. From the Figures 7–9, we can see that East Asia and Southeast Asia have been the active regions of the virus for their central role in the transmission of the virus. These areas happened to be the regions where several avian influenza A virus outbreaks recently started (Wallace et al., 2007; Rambaut et al., 2008; Russell et al., 2008; Li et al., 2014; Pollett et al., 2015).

The H9N2 virus was first discovered in USA, and then the virus “spread” to other areas of the world (Guo et al., 2000). The long-distance transmission of the virus in the Worldwide lineages implied a driven force come from the migratory birds, which may have carried the virus around seeding the virus to locations along their flyways. The fact that the transmission routes of the virus coincided with the main flyways of the wild birds further supported this assumption. While in the Asia-Africa and China lineages, the transmission routes seldom covered the bird flyways. The reason behind this was that the hosts of the virus in these lineages were mainly poultry such as chicken, duck, and quail. The migration distances of these hosts were shorter than those in the Worldwide lineages. Furthermore, people's specific lifestyle may also affect the ecology of the virus (Bedford et al., 2015). For example, the virus hosts like falcons and parakeets were found in the Middle East, where people domesticate falcons for pets, and train falcons to prey on poultry. In China, some people got used to buy live birds in the live poultry market and slaughter them at home for food. This tradition has recently been proven to have accelerated the spread of H7N9 virus in China (Bao et al., 2013). The Chinese government introduced polices to close live poultry markets in the epidemic hot regions, which has efficiently controlled the spread of the virus. Furthermore, slaughter of poultry carrying H9N2—the incubators for wild-bird-origin influenza viruses—has been an effective strategy to prevent human beings from becoming infected with avian influenza.

It should be noted that the transmission of all the three lineages of H9N2 virus covered the geographical location of mainland China, which implied a crucial role of this hot area for spreading the virus (Li et al., 2003). However, the H9N2 virus from the Worldwide lineages and Asia-Africa lineages did not become dominant variants in China. A selective sweep of the virus may have been carried out in this area, leaving those with higher fitness to circulate in the hosts which were mainly poultry. It has been reported that both the newly emerged H7N9 and H10N8 viruses in China possess internal gene cassettes recruited from poultry H9N2 virus (To et al., 2014; Jin et al., 2017). Continuing surveillance of H9N2 virus in poultry in China, especially the live poultry market, would benefit the control of potential new avian influenza A virus infecting human.

## Author contributions

LL, JY, and HR: formulated the study; MH and YJ: performed the research; JZ and ZH: analyzed the data; BL and WZ: participated in analysis and discussion; MH, HR, and JY: drafted the manuscript; All authors read and approved the final manuscript.

### Conflict of interest statement

The authors declare that the research was conducted in the absence of any commercial or financial relationships that could be construed as a potential conflict of interest.

## Footnotes

^1^Peden, J. *Codon W version 1.4.2*. Available online at: http://codonw.sourceforge.net/

## Supplementary material

Supplementary Table 1 The GI numbers of H9N2 sequences of all lineages.

Supplementary Table 2 The migration ways with Bayesian stochastic search variable selection tests of the HA Worldwide lineage.

Supplementary Table 3 The migration ways with Bayesian stochastic search variable selection tests of the NA Worldwide lineage.

Supplementary Table 4 The migration ways with Bayesian stochastic search variable selection tests of the HA Asia-Africa lineage.

Supplementary Table 5 The migration ways with Bayesian stochastic search variable selection tests of the NA Asia-Africa lineage.

Supplementary Table 6 The migration ways with Bayesian stochastic search variable selection tests of the HA China lineage.

Supplementary Table 7 The migration ways with Bayesian stochastic search variable selection tests of the NA China-I lineage.

Supplementary Table 8 The migration ways with Bayesian stochastic search variable selection tests of the NA China-II lineage.

Supplementary Table 9 The positive selection results of HA lineages.

Supplementary Table 10 The positive selection results of NA lineages.
